# Hemorrhagic Renal Angiomyolipoma in Pregnancy Effectively Managed by Immediate Cesarean Section and Elective Transcatheter Arterial Embolization: A Case Report

**DOI:** 10.1089/cren.2016.0030

**Published:** 2016-04-01

**Authors:** Satoru Kira, Norifumi Sawada, Tatsuya Miyamoto, Takahiko Mitsui, Hidenori Zakoji, Masayuki Takeda

**Affiliations:** Department of Urology, Interdisciplinary Graduate School of Medicine and Engineering, University of Yamanashi, Chuo, Japan.

## Abstract

Renal angiomyolipoma (AML) is a benign renal tumor with a risk of rupture in intratumoral aneurysms. Although renal AML in pregnancy is rare, risk of rupture is greater. Management for AML and childbirth is important during pregnancy; however, it is undefined yet. We present a case of hemorrhagic angiomyolipoma in pregnancy that is effectively managed by immediate cesarean section and elective transcatheter arterial embolization.

## Introduction and Background

Renal angiomyolipoma (AML) is an uncommon benign mesenchymal tumor composed of blood vessels, smooth muscle, and mature adipose tissue. Renal AML can spontaneously rupture and cause retroperitoneal hemorrhage. Although renal AML in pregnancy is relatively rare, due to changes in blood flow and pressure, compression by the enlarged uterus, and hormonal influence, it has a greater risk of rupture, consequently leading to medical emergency such as maternal shock or fetal death. Therefore, it is difficult to establish therapeutic strategies to manage both AML rupture and safe delivery. We present the case of a hemorrhagic AML in pregnancy managed effectively by immediate cesarean section and elective transcatheter arterial embolization (TAE).

## Case Presentation

**FIG. 1.**
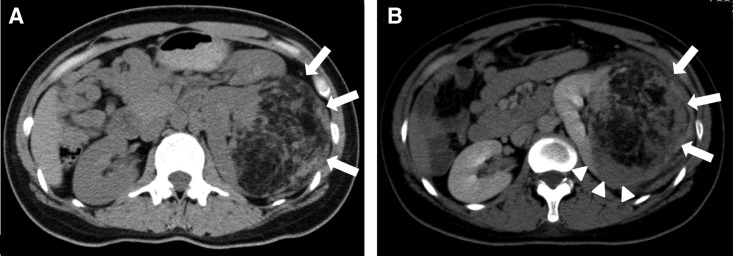
Computed tomography (CT) findings. **(A)** CT showed a ruptured left renal angiomyolipoma (AML; *arrows*) with a maximum diameter of 12 cm. **(B)** Enhanced CT showed a reruptured AML (*arrows*) with intratumoral hemorrhage (*arrowheads*).

A 30-year-old woman in her first 19th week of pregnancy with asymptomatic gross hematuria was referred to our department for the treatment of a left renal mass 12 cm in diameter, which was confirmed by computed tomography (CT) in a previous hospital (Fig. 1A). Gross hematuria stopped upon admission, and general condition of both the patient and fetus was good. Laboratory tests showed a hemoglobin level of 11.2 g/dL, and urinalysis test results, including urine cytology, were all negative. The patient had no significant medical history, hereditary disease such as tuberous sclerosis, or recognized common genetic disorders. CT, MRI, and color Doppler ultrasonography findings showed a left renal AML containing a fat component and some aneurysms. Considering these findings, we concluded that a minor rupture of the AML was responsible for the gross hematuria. We discussed a treatment strategy with an obstetrician and interventional radiologist and decided for a conservative management with a planned cesarean section at 36 weeks of pregnancy. If AML was reruptured during pregnancy up to cesarean section, we would consider and prepare emergent TAE for treatment. Therefore, the patient was instructed to rest at home and avoid vigorous exercise as much as possible. A close follow-up was planned for immediate intervention in case of bleeding of aneurysm rupture in AML. At 34 weeks of pregnancy, the patient presented to our hospital with a left back pain. A CT findings showed retroperitoneal hemorrhage caused by a reruptured AML aneurysm (Fig. 1B). Laboratory tests showed a hemoglobin level of 9.7 g/dL.

## Intervention

**FIG. 2.**
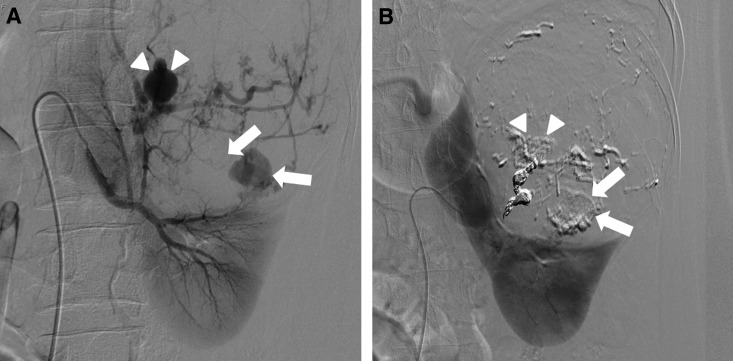
Angiography findings. **(A)** Angiography revealed two aneurysms. The ruptured aneurysm had an irregular shape (*arrows*) and the other one had a regular shape (*arrowheads*). **(B)** Arteries leading toward each aneurysm were embolized.

At the time of the consultation, the patient and fetus were hemodynamically stable. However, considering the AML rerupture, we decided to perform an immediate cesarean section earlier than the previously scheduled date and then a selective TAE to treat the reruptured AML. A healthy infant was delivered by a cesarean section, which was followed by an angiography and selective TAE. Angiography showed two AML feeding arteries and two aneurysms, one on each of the arteries (Fig. 2A). Although one aneurysm had an irregular shape, which was responsible for bleeding, and the other regular, there was no contrast extravasation clearly from either aneurysm. All aneurysms were embolized with gelatin sponge particles and coils (Fig. 2B). Both the patient and the baby were discharged uneventfully 7 days after the procedures.

## Discussion and Literature Review

The most severe problem related to renal AML is spontaneous hemorrhage. Because it is minimally invasive and also provides an opportunity for renal preservation, TAE has been the standard intervention for treatment of AML rupture and prevention of rerupture.^1^ These benefits are even more important for management of AML in pregnancy. However, only a few cases of AML rupture during pregnancy treated with TAE were reported so far. When considering the potential risk of carcinogenesis, the radiation exposure to the fetus must be minimized. Furthermore, a consensus on the modifications of TAE-related dose limits for radiation exposure when treating AML in pregnancy has not yet been reached. Recently it has been suggested that the decision to perform TAE should be based on hemodynamic stability of the mother and fetus.^2^ If they are stable, conservative management including close follow-up or elective TAE is considered first. In our case, we decided for a conservative management of the first AML rupture, because hemorrhage was not significant and both the patient and fetus were hemodynamically stable. However, when the AML rerupture occurred, we decided for invasive management of both the hemorrhagic AML and pregnancy. At that time, hemodynamic stability in both the patient and the fetus was recognized. However, CT findings clearly showed a retroperitoneal hemorrhage caused by an AML rupture. Furthermore, the pregnancy was close to the date of the scheduled elective cesarean section. Thus, considering several factors including the hemodynamic stability of mother and fetus, number of weeks in pregnancy (possibility of cesarean section), and the extent of hemorrhage, active treatment for ruptured AML should be considered in case of reruptured AML in pregnancy.

## Abbreviations Used

AML: angiomyolipoma
CT: computed tomography
TAE: transcatheter arterial embolization

## References

[1] Flum AS, Hamoui N, Said MA, et al. Update on the diagnosis and management of renal angiomyolipoma. J Urol 2015 [Epub ahead of print]; DOI: 10.1016/j.juro.2015.07.126 26612197

[2] Myoen S, Mitsuzuka K, Saito H, et al. Spontaneous rupture of a renal angiomyolipoma at 25 weeks of pregnancy treated with transarterial embolization: A case report and review of the literature. Int J Urol 2015;22:710–712 25881870 10.1111/iju.12775

